# Machine learning prediction of right heart failure after left ventricular assist device implantation with individual risk factor visualization

**DOI:** 10.1016/j.xjon.2026.101728

**Published:** 2026-03-19

**Authors:** Takaaki Samura, Akane Musha, Daisuke Yoshioka, Kohei Tonai, Takuji Kawamura, Ai Kawamura, Yusuke Misumi, Takuya Watanabe, Takuma Sato, Arisa Ikeda, Yukiko Endo, Hideki Masaki, Hiromitsu Nakauchi, Hidetsugu Asanoi, Yasumasa Tsukamoto, Satsuki Fukushima, Yasushi Sakata, Shigeru Miyagawa

**Affiliations:** a Department of Cardiovascular Surgery, Osaka University Graduate School of Medicine, Osaka, Japan; b Stem Cell Therapy Division, Institute of Integrated Research, Institute of Science Tokyo, Tokyo, Japan; c Institute of Stem Cell Biology and Regenerative Medicine, Stanford University School of Medicine, Stanford, Calif; d Department of Cardiovascular Surgery, National Cerebral and Cardiovascular Center, Osaka, Japan; e Department of Transplantation, National Cerebral and Cardiovascular Center, Osaka, Japan; f Department of Cardiology, Osaka University Graduate School of Medicine, Osaka, Japan

**Keywords:** right heart failure, left ventricular assist device, machine learning, SHAP value analysis

## Abstract

**Background:**

Right ventricular failure (RVF) is a major adverse event after left ventricular assist device (LVAD) implantation, but its complex mechanisms make accurate prediction challenging. While supervised machine learning (ML) can predict outcomes from complex models, understanding the conclusions is often difficult. This study aimed to develop an interpretable supervised ML model to assess patients’ RVF risk and identify key risk factors.

**Methods:**

We analyzed 327 patients from 482 consecutive LVAD implantations performed between June 2010 and January 2024 who underwent preoperative right heart catheterization and echocardiography. RVF was defined as requiring a right ventricular assist device or ≥14 days of postoperative inotropic support. Preoperative clinical and hemodynamic variables were used for risk prediction, and Shapley additive explanation (SHAP) values were used to assess individual risk factors.

**Results:**

Thirteen features were selected: sex, age, nonischemic cardiomyopathy, body surface area, aspartate aminotransferase, blood urea nitrogen, left ventricular end-diastolic dimension, left ventricular ejection fraction, cardiac output, right ventricular stroke work index, central venous pressure (CVP)/pulmonary capillary wedge pressure (PCWP), pulmonary pulsatility index (PAPi), and Interagency Registry for Mechanically Assisted Circulatory Support (INTERMACS) profile. Ensemble learning was the most reliable algorithm, achieving an area under the curve of 0.888 (95% confidence interval [CI], 0.756-0.975) and an accuracy of 0.879 (95% CI, 0.788-0.940). A high-risk group (RVF occurrence rate >20%) had 1-year and 3-year survival rates of 80.7% and 75.3%, respectively, showing a tendency toward worse long-term outcomes compared to the low-risk group (93.2% vs 90.5%; *P* = .09). SHAP analysis revealed individual patient risk factors and their contributions, most frequently highlighting CVP/PCWP, PAPi, and INTERMACS profile.

**Conclusions:**

Supervised ML enables accurate prediction of RVF after LVAD implantation. SHAP values identify individual risk factors, potentially optimizing preoperative conditions.

**Figure:**
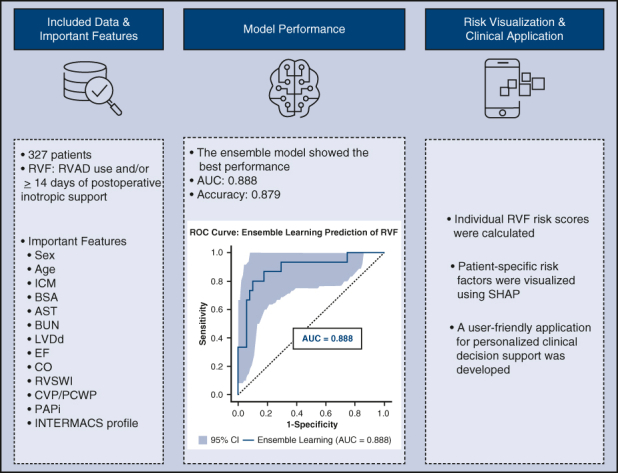
Using data from 327 LVAD patients from 2 high-volume centers in Japan, we developed a machine learning model to predict postoperative right ventricular failure. The ensemble model achieved the best performance (AUC = 0.888) and enables individualized risk estimation and SHAP-based visualization of patient-specific risk factors in a user-friendly clinical decision support application.

**Central Message**

Machine learning models accurately predict RVF post-LVAD. Using SHAP values, our model visualizes patient-specific risk factors, offering insights that can optimize preoperative conditions and guide clinical decisions.

**Perspective**

Supervised machine learning models can accurately predict RVF after LVAD implantation. By using SHAP values to visualize each patient’s key risk factors, we can support the interpretability of machine learning models. Clinicians can then act on these insights to personalize preoperative optimization, ultimately improving patient outcomes.

Continuous-flow left ventricular assist devices (LVADs) are being increasingly used to treat patients with end-stage heart failure^1^^,^^2^; however, right ventricular failure (RVF) is a common and serious complication that significantly increases perioperative mortality rates following LVAD implantation. Previous studies have reported a prevalence of RVF following LVAD implantation ranging from 10% to 40%.^3^, ^4^, ^5^, ^6^, ^7^ RVF following LVAD implantation is associated with poor outcomes.^7^^,^^8^ Numerous previous studies have identified clinical predictors of RVF based on hemodynamic parameters including elevated central venous pressure (CVP), CVP/pulmonary capillary wedge pressure (PCWP) ratio, decreased right ventricular stroke work index (RVSWI), and pulmonary artery pulsatility index (PAPi).^5^^,^^9^, ^10^, ^11^ However, such studies often relied on regression models to analyze the relationship between baseline variables and outcomes. Although useful, these models have a limited ability to account for complex interactions among baseline variables.

Supervised machine learning (ML) recently has demonstrated remarkable success in classification and prediction, even in clinical research.^12^, ^13^, ^14^ ML has the potential to more accurately predict outcomes by considering the interactions between baseline variables; however, it often is perceived as a “black box” involving an unclear decision making process. In the present study, we aimed to develop an interpretable supervised ML model to predict the individual risk of RVF after LVAD implantation and to identify unique risk factors.

## Methods

### Patients

**Figure 1:**
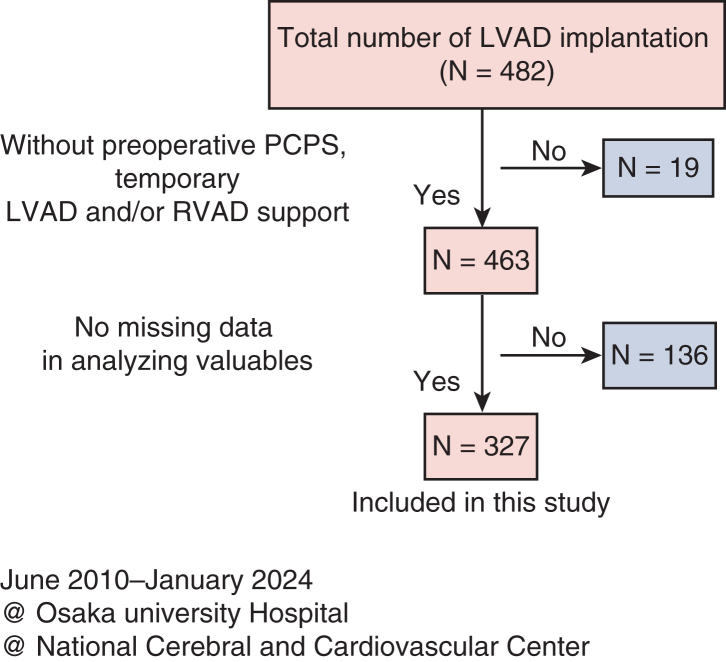
Patient selection algorithm used in the study. *LVAD*, Left ventricular assist device; *RVAD*, right ventricular assist device.

A database search at Osaka University Hospital and the National Cerebral and Cardiovascular Center identified patients who met the following criteria: (1) use of a continuous-flow LVAD as the primary implant and age >18 years; (2) no preoperative extracorporeal membranous oxygenation and/or extracorporeal LVAD or right ventricular assist device support; and (3) absence of missing data in the analyzed variables. Ultimately, the data of 327 patients were included in our analyses (Figure 1). All clinical data were analyzed in an anonymized manner. RVF was defined as requiring a right ventricular assist device, ≥14 days of postoperative inotropic support, or both.

All patients and their family members provided informed consent for publication prior to undergoing LVAD implantation. This study was approved by the Human Subjects Review Committees of Osaka University Hospital and National Cerebral and Cardiovascular Center (approval R21114-2; approved March 28, 2024).

### Devices and Medical Management After LVAD Implantation

The LVAD type used for each patient was determined during the preoperative conference because the availability of these devices in Japan has changed over the years. In brief, the DuraHeart (Terumo Heart) and EVAHEART (Sun Medical) were approved in October 2011, the HeartMate II (Abbott Medical) was approved in April 2013, the HeartWare (Medtronic) and Jarvik 2000 (Jarvik Heart) were approved in May 2014, and the HeartMate 3 (Abbott Medical) was approved in December 2020. Following LVAD implantation, all patients received a standardized heart failure management regimen that included neurohormonal antagonists, diuretics, and antiarrhythmic agents as clinically indicated.

### Data Collection

The patients’ preoperative data were obtained retrospectively from their electronic medical records: baseline demographics, medical history, blood chemistry, echocardiographic parameters, and hemodynamic parameters obtained via right heart catheterization. According to previous studies, the RVSWI, pulmonary vascular resistance (PVR), and PAPi were calculated as follows: RVSWI = (mean PAP – mean RAP) × stroke volume index^9^; PVR = (mean PAP – PCWP)/CO; and PAPi = (systolic PAP – diastolic PAP)/RAP.^10^

### Selection of Important Features

All statistical analyses and classifications were performed using JMP 16.0 (SAS Institute) and custom programs written in Python 3.12.3. Categorical variables are summarized as frequency and percentage and were compared between groups using the χ^2^ test. Continuous variables are summarized as mean ± standard deviation or median (interquartile range) and were compared using the Mann-Whitney *U* test. Survival rates were estimated using Kaplan-Meier curves.

Regarding the selection of important features, baseline characteristics, laboratory values, and hemodynamic parameters were included in this analysis. We selected informative valuables using the χ^2^ or Mann-Whitney *U* test and the Gini index in a random forest algorithm, and variables deemed essential based on a literature review were added to ensure the inclusion of clinically significant features. For the χ^2^ test and Mann-Whitney *U* test, significance was set at *P* < .10. If this condition was not met, the Gini index in a random forest algorithm was used to compare the significance of similar features. Features identified as important based on the literature review and patient baseline information also were considered. Moreover, to minimize multicollinearity, candidate predictors were prescreened using Spearman's rank correlation analysis; variables with strong correlations (|ρ| ≥ 0.8) were excluded only when they were considered medically overlapping.

### Classification Algorithm

**Figure 2:**
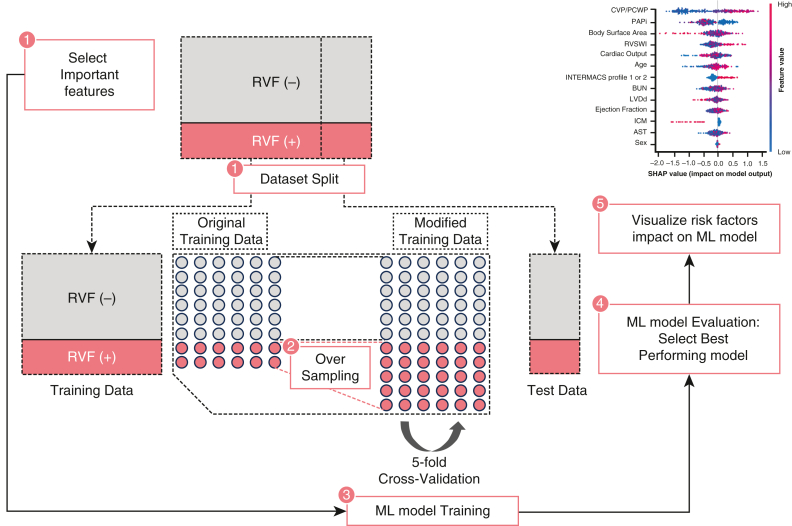
Overview of the machine learning (ML) process. *RVF*, Right ventricular failure.

The following 5-step process was implemented for ML:

(1) Selection of important features and removal of missing data. Important features were selected and missing data excluded. Because the data were imbalanced, the training and test datasets were split at an 8:2 ratio to ensure an equal proportion of positive cases.
(2) Oversampling of training data. This was done to create new synthetic positive samples, thereby increasing the number of positive cases for constructing the predictive model.
(3) Cross-validation and parameter tuning. Using the augmented training data, a 5-fold cross-validation was conducted, and the parameters were tuned based on the F-score.
(4) Evaluation of multiple ML models. The test data were used to evaluate multiple ML models, and the best-performing model was selected.
(5) Visualization of risk factors for RVF in each case using Shapley additive explanations (SHAP) value analysis of test data. SHAP values were calculated for the test data to identify which factors contributed to the RVF prediction in each case (Figure 2).^15^

During the training data oversampling process, different oversampling methods, including ADASYN, SMOTEENN, and SMOTE, were compared.^16^^,^^17^ In the cross-validation process, hyperparameter tuning and optimization were performed for each ML algorithm, including random forest, eXtreme Gradient Boosting (XGBoost), support vector machines (SVM), logistic regression, and ensemble learning.

The ML models calculated a probability score of RVF, which was then used to determine whether individual patients would develop RVF. The test data were evaluated by comparing the models based on the mean area under the curve (AUC), prediction accuracy, positive predictive value, and F-score. The F-score was calculated as F-score = $2\times \frac{PPV\times Sensitivity}{PPV+Sensitivity}$, while the SHAP score was used to assess the individual risk factors for RVF. To evaluate the stability of the model performance, we performed 1000 iterations of bootstrap resampling to calculate the 95% confidence intervals for the AUC, accuracy, and other evaluation metrics, allowing for robust estimation of predictive performance. By analyzing the relationship between the probability score and the actual RVF rate in the test data, we derived a locally estimated scatterplot smoothing (LOESS) function to calibrate the raw model scores into estimated clinical risk probabilities. This nonparametric approach was used to ensure that the output scores more accurately reflect the observed incidence of RVF across the entire score range.

## Results

### Baseline Characteristics and Incidence of RVF

A total of 327 patients met the inclusion criteria (Table 1). The mean patient age was 46 ± 13 years, and 98 patients (30%) were female. Idiopathic dilated cardiomyopathy was observed in 196 patients (60%), dilated hypertrophic cardiomyopathy in 52 (16%), and ischemic cardiomyopathy (non-ICM) in 31 (9%). At LVAD implantation, 26% (n = 84) had an Interagency Registry for Mechanically Assisted Circulatory Support (INTERMACS) profile of 1 or 2. During the post-LVAD hospital stay, 72 patients (22%) experienced RVF.

**Table 1:** Patient characteristics before LVAD implantation (n = 327)

Characteristic	Value
Age at LVAD implantation, y, mean ± SD	46 ± 13
Female sex, n (%)	98 (30)
Body surface area, m^2^, mean ± SD	1.6 ± 0.2
Body mass index, mean ± SD	20.5 ± 3.3
Etiology, n (%)	
Ischemic cardiomyopathy	31 (9)
Idiopathic dilated cardiomyopathy	196 (60)
Dilated phase of hypertrophic cardiomyopathy	52 (16)
Preoperative hemodynamics, n (%)	
Inotropes	76 (23)
Intra-aortic balloon pumping	43 (13)
Intubation	9 (3)
INTERMACS profile 1 or 2	84 (26)
Preoperative right heart catheterization parameters, mean ± SD	
Heart rate, bpm	80 ± 15
CVP, mmHg	7.0 ± 4.4
Mean PAP, mmHg	27.8 ± 10.5
PWCP, mmHg	19.4 ± 8.7
Cardiac index, L/min/m^2^	2.0 ± 0.5
PVR, Wood units	2.8 ± 1.8
RVSWI	518 ± 253
PAPi	4.2 ± 3.4
CVP/PCWP	0.39 ± 0.26
Preoperative echocardiographic parameters, mean ± SD	
Left ventricular end-diastolic dimension, mm	70.2 ± 12.9
Left ventricular end-systolic dimension, mm	64.3 ± 13.6
Left ventricular ejection fraction, %	20.1 ± 8.6
Preoperative laboratory valuables, median (IQR)	
White blood cell count, cells/mm^3^	6000 (4635-7265)
Hemoglobin, g/dL	11.8 (10.7-13.3)
Serum albumin, g/dL	3.9 (3.5-4.2)
BUN, mg/dL	17 (13-22)
Serum creatinine, mg/dL	0.97 (0.78-1.22)
Aspartate transaminase, IU/dL	23 (19-29)
Alanine transaminase, IU/dL	19 (14-29)
Total bilirubin, mg/dL	0.8 (0.6-1.2)
Brain natriuretic peptide, pmol/L	436 (236-806)
Devices	
Axial type of LVAD, n (%)	186 (57)

*LVAD*, Left ventricular assist device; *SD*, standard deviation; *INTERMACS*, Interagency Registry for Mechanically Assisted Circulatory Support; *RHC*, right heart catheterization; *CVP*, central venous pressure; *PAP*, pulmonary artery pressure; *PCWP*, pulmonary capillary wedge pressure; *PVR*, pulmonary vascular resistance; *RVSWI*, right ventricular stroke work index; *PAPi*, pulmonary artery pulsatility index; *IQR*, interquartile range; *BUN*, blood urea nitrogen.

### Variable Importance

**Figure E1:**
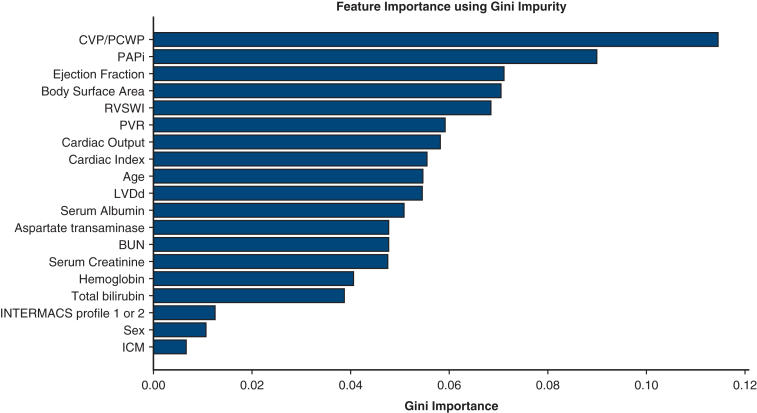
Gini importance of features in the dataset. *BUN*, Blood urea nitrogen; *CVP*, central venous pressure; *ICM*, ischemic cardiomyopathy; *LVDd*, left ventricular end-diastolic dimension; *PAPi*, pulmonary artery pulsatility index; *PCWP*, pulmonary capillary wedge pressure; *PVR*, pulmonary vascular resistance; *RVSWI*, right ventricular stroke work index.

**Figure E2:**
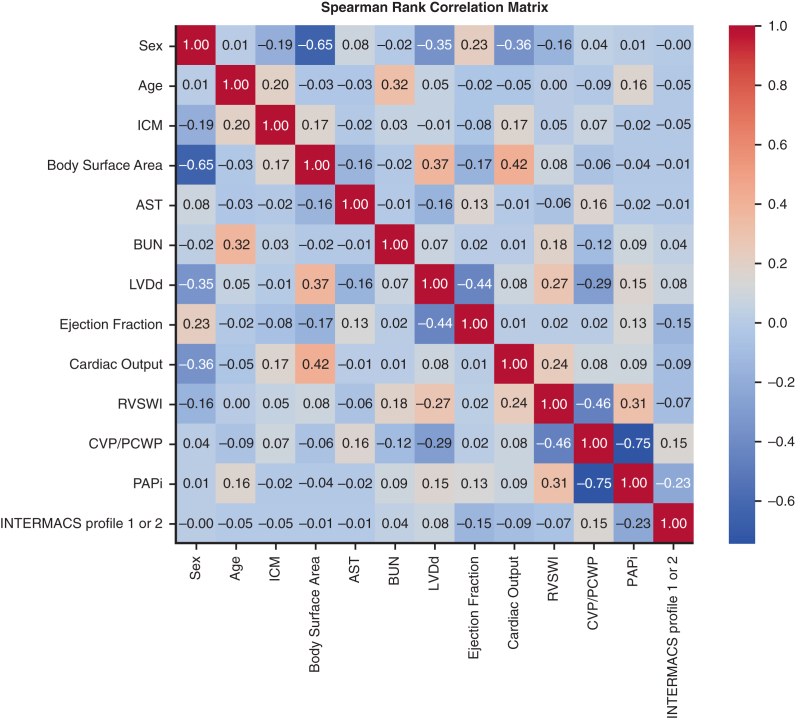
Correlation matrix of clinical variables included in the model. *BUN*, Blood urea nitrogen; *CVP*, central venous pressure; *ICM*, ischemic cardiomyopathy; *LVDd*, left ventricular end-diastolic dimension; *PAPi*, pulmonary artery pulsatility index; *PCWP*, pulmonary capillary wedge pressure; *PVR*, pulmonary vascular resistance; *RVSWI*, right ventricular stroke work index.

The χ^2^ or Mann-Whitney *U* test revealed that body surface area (BSA), non-ICM, preoperative use of inotropes, intra-aortic balloon pump implantation, intubation, an INTERMACS profile 1 or 2, CVP, CVP/PCWP, PAPi, and left ventricular end-diastolic dimension (LVDd) were significant, with values of *P* < .1 (Table 2). Regarding the preoperative condition, among the patients with an INTERMACS profile 1 or 2, the related preoperative uses of inotropes, intra-aortic balloon pumping, and intubation were reduced. Regarding preoperative hemodynamics, *P* values and the Gini index were also considered, and related factors were reduced to select CVP/PCWP, PAPi, RVSWI, and cardiac output. For the echocardiographic data, ejection fraction was added based on the Gini index, and LVDd and ejection fraction were selected. Based on the literature review, preoperative patient baseline information, renal function, and liver function were considered, and age, sex, blood urea nitrogen (BUN), and aspartate transaminase (AST) were added based on the χ^2^ test, Mann-Whitney *U* test, and Gini index (Figure E1). Consequently, 13 medically significant features suitable for prediction were selected. Subsequent correlation analysis of the selected variables revealed no excessive pairwise correlations, indicating that the final feature set did not exhibit problematic multicollinearity (Figure E2).

**Table 2:** Factors associated with RVF (N = 327)

Factor	RVF-negative (N = 255)	RVF-positive (N = 72)	*P* value
Age at LVAD implantation, y, mean ± SD	46 ± 13	48 ± 12	.223
Female sex, n (%)	73 (29)	25 (35)	.329
Body surface area, m^2^, mean ± SD	1.64 ± 0.18	1.59 ± 0.18	.077
Body mass index, mean ± SD	20.7 ± 3.3	20.0 ± 3.2	.157
Etiology, n (%)			
Ischemic cardiomyopathy	29 (11)	2 (3)	.027
Idiopathic dilated cardiomyopathy	153 (60)	43 (60)	.937
Dilated phase of hypertrophic cardiomyopathy	39 (15)	13 (18)	.581
Preoperative hemodynamics, n (%)			
Inotropes	188 (74)	62 (86)	.032
Intra-aortic balloon pump	24 (9)	19 (26)	<.001
Intubation	2 (1)	7 (9)	<.001
INTERMACS profile 1 or 2	56 (22)	28 (39)	.004
Preoperative right heart catheterization parameters, mean ± SD			
Heart rate, bpm	79 ± 15	80 ± 14	.388
CVP, mmHg	6.4 ± 4.1	9.2 ± 5.1	<.001
Mean PAP, mmHg	26.3 ± 10.5	27.3 ± 9.5	.230
PWCP, mmHg	19.4 ± 8.9	19.2 ± 7.9	.934
Cardiac index, L/min/m^2^	2.0 ± 0.5	2.0 ± 0.6	.865
Cardiac output, L/min	3.3. ± 1.0	3.2 ± 1.0	.463
Pulmonary vascular resistance (Wood)	2.3 ± 1.7	2.8 ± 2.0	.115
RVSWI	494 ± 244	456 ± 249	.212
PAPi	4.5 ± 3.5	3.0 ± 2.7	<.001
CVP/PCWP	0.36 ± 0.24	0.51 ± 0.29	<.001
Preoperative echocardiographic parameters, mean ± SD			
Left ventricular end-diastolic dimension, mm	70.9 ± 13.0	67.6 ± 12.2	.095
Left ventricular end-systolic dimension, mm	65.0 ± 13.6	61.7 ± 13.7	.139
Left ventricular ejection fraction, %	19.6 ± 8.0	21.7 ± 10.3	.109
Preoperative laboratory valuables, median (IQR)			
White blood cell count, mm^3^	5950 (4685-7215)	6105 (4545-7508)	.879
Hemoglobin, g/dL	11.9 (10.7-13.3)	11.8 (10.2-13.2)	.359
Serum albumin, g/dL	3.9 (3.5-4.2)	3.9 (3.3-4.2)	.638
BUN, mg/dL	17 (13-22)	18.5 (14-23)	.437
Serum creatinine, mg/dL	0.97 (0.78-1.22)	0.97 (0.78-1.19)	.909
Aspartate aminotransferase, IU/dL	23 (18-28)	24 (20-32)	.108
Alanine transaminase, IU/dL	19 (14-28)	20 (13-30)	.957
Total bilirubin, mg/dL	0.8 (0.6-1.2)	0.8 (0.6-1.2)	.315
Brain natriuretic peptide, pmol/L	417 (230-799)	509 (271-857)	.321
Devices			
Axial type of LVAD, n (%)	143 (56)	43 (60)	.605

*RVF*, Right ventricular failure; *LVAD*, left ventricular assist device; *SD*, standard deviation; *INTERMACS*, Interagency Registry for Mechanically Assisted Circulatory Support; *RHC*, right heart catheterization; *CVP*, central venous pressure; *PAPi*, pulmonary artery pulsatility index; *PCWP*, pulmonary wedge capillary pressure; *RVSWI*, right ventricular stroke work index; *IQR*, interquartile range; *BUN*, blood urea nitrogen.

### Predictive Performance

**Figure 3:**
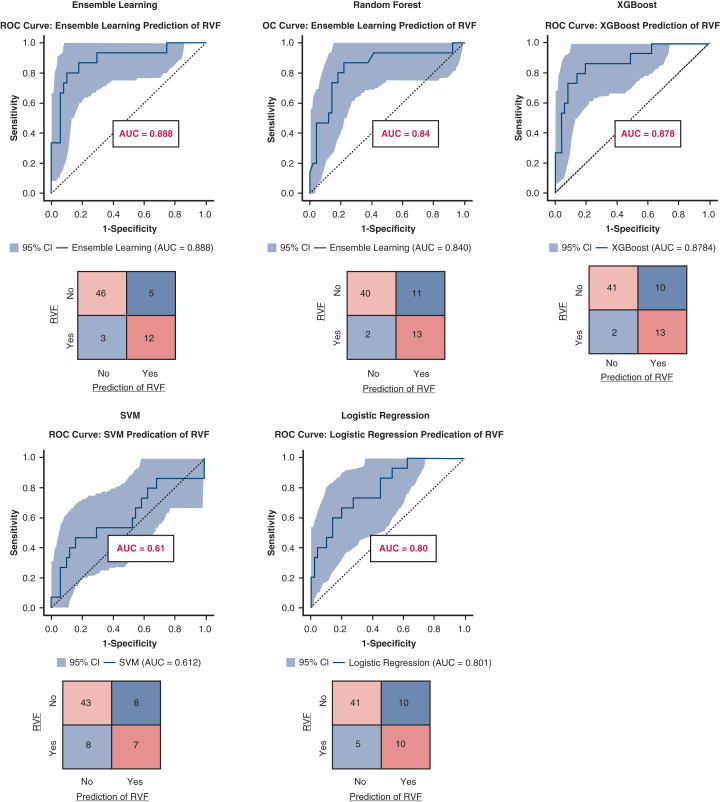
Prediction of right ventricular failure (RVF) after left ventricular assist device (LVAD) implantation. Ensemble learning demonstrated a superior area under the curve (AUC) compared to other methods. *SVM*, Support vector machines; *XGBoost*, eXtreme Gradient Boosting.

The AUC, accuracy, F1 score, and sensitivity of the random forest, XGBoost, SVM, logistic regression, and ensemble learning algorithms were analyzed using the 13 selected variables (Figure 3, Table E1). Ensemble learning demonstrated superior performance for predicting RVF; specifically, its performance metrics were as follows (values in parentheses indicate 95% confidence intervals [CI]): AUC, 0.888 (0.756-0.975); accuracy, 0.879 (0.788-0.940); F1 score, 0.750 (0.546-0.889); and sensitivity, 0.800 (0.579-1.000).

### Visualization of Individual Risk Factors

**Figure E3:**
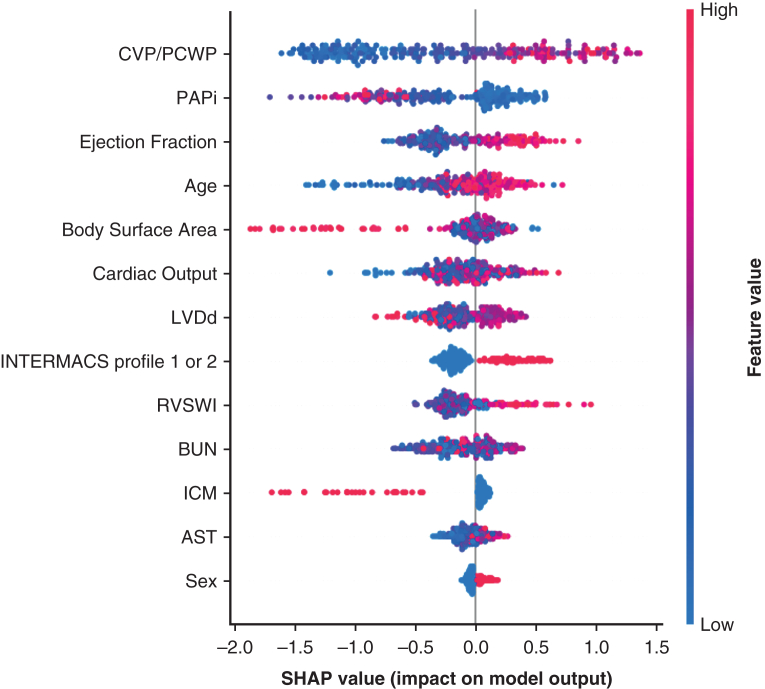
Shapley additive explanations (SHAP) values of each important valuable in the training data. Larger numerical values are plotted in red. The horizontal axis indicates the SHAP value, with larger values to the right contributing more to right ventricular failure. *AST*, Aspartate transaminase; *BUN*, blood urea nitrogen; *CVP*, central venous pressure; *ICM*, ischemic cardiomyopathy; *LVDd*, left ventricular end-diastolic dimension; *PAPi*, pulmonary artery pulsatility index; *PCWP*, pulmonary capillary wedge pressure; *RVSWI*, right ventricular stroke work index.

The SHAP values clarified each factor’s contribution to the training data (Figure E3), as well as its influence on the individual predictions (Table 3). A positive SHAP value indicated an increased risk of RVF, whereas a negative SHAP value suggested a reduced risk. An analysis of the training data demonstrated that an elevated CVP/PCWP and reduced PAPi were associated with a higher risk of RVF, indicating that right ventricular function alone was the primary influencing factor. Table 3 presents the factors identified as risk or protective in the 17 cases in which the ML algorithms predicted a high risk of RVF in the test data. A high CVP/PCWP, low PAPi, INTERMACS profile of 1 or 2, and low BSA were frequently observed. In contrast, normal liver and kidney function were identified as protective factors.

**Table 3:** High risk of RVF predicted by machine learning in test data

Case	Important features													Probability score	Individual SHAP value						Actual positive
	Sex	Age, y	ICM	BSA, m^2^	AST, IU/dL	BUN, mg/dL	LVDd, mm	EF, %	CO, L/min	RVSWI	CVP/PCWP	PAPi	INTERMACS 1 or 2		Risk factor 1	Risk factor 2	Risk factor 3	Protective factor 1	Protective factor 2	Protective factor 3	
1	M	59	No	1.66	36	32	71	7	4.70	0.76	0.33	2.38	Yes	0.646	RVSWI (0.515)	LVDd (0.332)	PAPi (0.233)	EF (−0.520)	CVP/PCWP (−0.489)	BUN (−0.294)	Yes
2	F	52	No	1.33	18	77	72	20	3.44	0.03	1.14	1.19	No	0.809	CVP/PCWP (1.138)	PAPi (0.410)	CO (0.224)	EF (−0.347)	INTERMACS 1 or 2 (−0.242)	BUN (−0.217)	No
3	M	66	No	1.59	26	17	79	11	2.95	0.71	0.47	1.93	Yes	0.826	CVP/PCWP (0.819)	INTERMACS 1 or 2 (0.606)	PAPi (0.316)	BUN (−0.400)	EF (−0.193)	AST (−0.159)	Yes
4	F	37	No	1.53	52	14	64	23	2.82	0.33	0.42	2.20	No	0.765	CVP/PCWP (0.585)	PAPi (0.411)	EF (0.312)	Age (−0.312)	LVDd (−0.178)	CO (−0.142)	Yes
5	F	34	No	1.43	24	8	57	36	2.57	0.06	1.00	0.63	Yes	0.783	CVP/PCWP (1.138)	EF (0.378)	PAPi (0.237)	Age (−0.567)	CO (−0.249)	BUN (−0.132)	No
6	M	43	No	1.54	30	9	47	46	4.01	0.31	0.62	2.25	No	0.648	CVP/PCWP (0.604)	EF (0.535)	PAPi (0.291)	BUN (−0.300)	LVDd (−0.290)	INTERMACS 1 or 2 (−0.233)	Yes
7	F	44	No	1.27	19	14	48	34	2.19	0.22	0.80	0.81	No	0.858	CVP/PCWP (1.222)	EF (0.372)	BSA (0.307)	LVDd (−0.230)	AST (−0.100)	INTERMACS 1 or 2 (−0.094)	Yes
8	M	51	No	1.82	29	21	69	32	2.82	0.48	0.58	1.73	No	0.863	CVP/PCWP (0.944)	PAPi (0.478)	EF (0.420)	INTERMACS 1 or 2 (−0.247)	CO (−0.214)	BSA (−0.106)	Yes
9	F	50	No	1.16	25	20	55	23	1.69	0.25	0.50	1.75	Yes	0.785	CVP/PCWP (0.874)	PAPi (0.311)	Age (0.308)	CO (−0.922)	LVDd (−0.186)	AST (−0.114)	Yes
10	F	63	No	1.62	20	16	67	12	2.12	0.35	0.52	1.82	Yes	0.809	CVP/PCWP (1.086)	INTERMACS 1 or 2 (0.615)	Age (0.273)	BUN (−0.464)	EF (−0.401)	LVDd (−0.215)	Yes
11	M	38	No	1.61	27	13	70	15	3.34	0.43	0.43	2.83	No	0.595	CVP/PCWP (0.626)	LVDd (0.306)	CO (0.291)	EF (−0.511)	BUN (−0.260)	INTERMACS 1 or 2 (−0.206)	No
12	F	19	No	1.67	22	8	44	59	3.53	0.29	0.67	2.50	No	0.579	CVP/PCWP (0.675)	EF (0.415)	CO (0.342)	Age (−1.129)	BUN (−0.249)	LVDd (−0.237)	Yes
13	F	59	No	1.44	32	33	69	13	2.36	0.22	0.82	2.33	No	0.742	CVP/PCWP (0.965)	CO (0.348)	PAPi (0.172)	EF (−0.427)	INTERMACS 1 or 2 (−0.230)	Age (−0.107)	Yes
14	M	48	No	1.73	16	12	86	13	2.93	0.46	0.50	2.33	No	0.589	CVP/PCWP (0.881)	PAPi (0.226)	Age (0.166)	EF (−0.352)	INTERMACS 1 or 2 (−0.296)	BUN (−0.271)	Yes
15	F	55	No	1.40	40	15	67	24	3.07	0.30	0.57	4.00	No	0.823	CVP/PCWP (1.195)	Age (0.329)	EF (0.325)	PAPi (−0.654)	INTERMACS 1 or 2 (−0.224)	RVSWI (−0.154)	No
16	M	61	No	1.40	22	18	57	22	3.67	0.82	0.57	2.88	Yes	0.839	INTERMACS 1 or 2 (0.548)	CVP/PCWP (0.542)	RVSWI (0.254)	BUN (−0.297)	LVDd (−0.168)	Sex (−0.050)	Yes

*RVF*, Right ventricular failure; *ICM*, ischemic cardiomyopathy; *BSA*, body surface area; *AST*, aspartate aminotransferase; *BUN*, blood urea nitrogen; *LVDd*, left ventricular end-diastolic dimension; *EF*, ejection fraction; *CO*, cardiac output; *RVSWI*, right ventricular stroke work index; *CVP*, central venous pressure; *PCWP*, pulmonary wedge capillary pressure; *INTERMACS*, Interagency Registry for Mechanically Assisted Circulatory Support; *PAPi*, pulmonary artery pulsatility index.

### RVF Risk and All-Cause Mortality in Test Data

**Figure 4:**
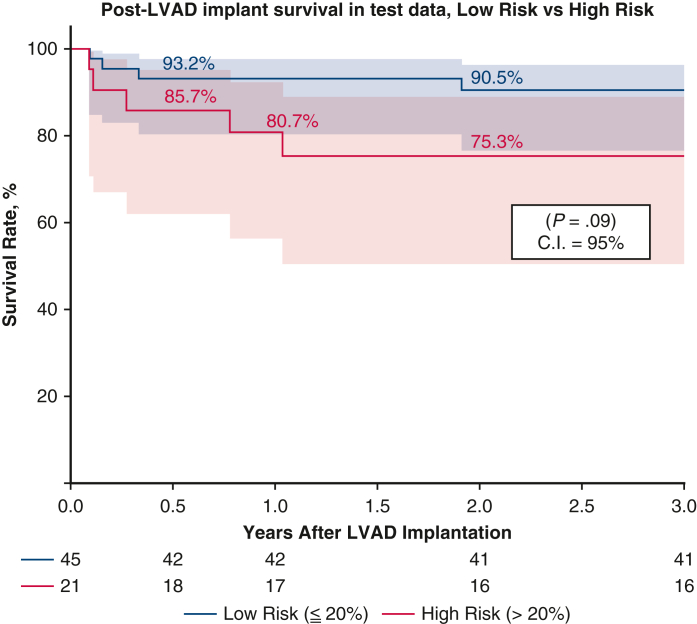
Post–left ventricular assist device (LVAD) implantation survival in test data, low risk versus high risk. The low-risk group tended to show better long-term outcomes (log-rank test, *P* = .09). Shaded area indicates 95% confidence interval.

**Figure E4:**
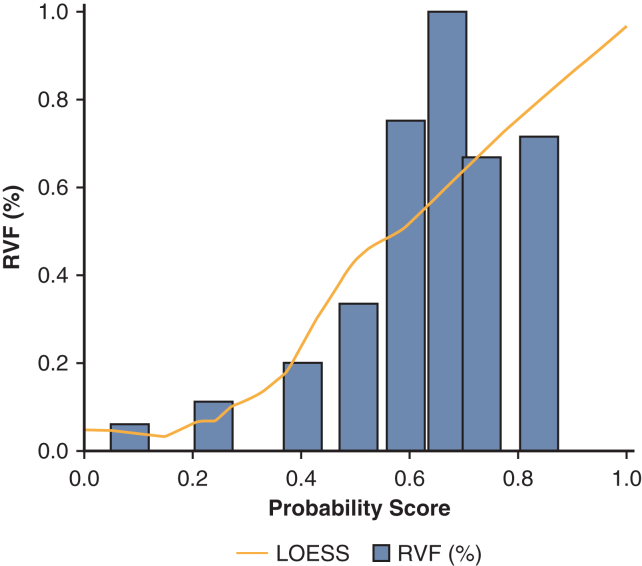
Relationship between probability score and right ventricular failure (RVF) rate in test data. The curve approximating the relationship between the probability score and RVF incidence was generated using a locally estimated scatterplot smoothing (LOESS) curve.

We calculated the predicted RVF occurrence rate from the probability score using a LOESS curve (Figure E4) and then investigated long-term outcomes in the test data based on different risk levels. We compared a low-risk group (RVF occurrence rate ≤20%) with a high-risk group (RVF occurrence rate >20%). The 0.5-, 1-, 2-, and 3-year survival rates were 93.2%, 93.2%, 90.5%, and 90.5%, respectively in the low-risk group and 85.7%, 80.7%, 75.3%, and 75.3%, respectively, in the high-risk group. While there was no statistically significant difference between the groups by the log-rank test (*P* = .09), the high-risk group showed a tendency toward worse long-term outcomes (Figure 4).

### Compared to the STOP-RVF Risk Calculator

**Figure E5:**
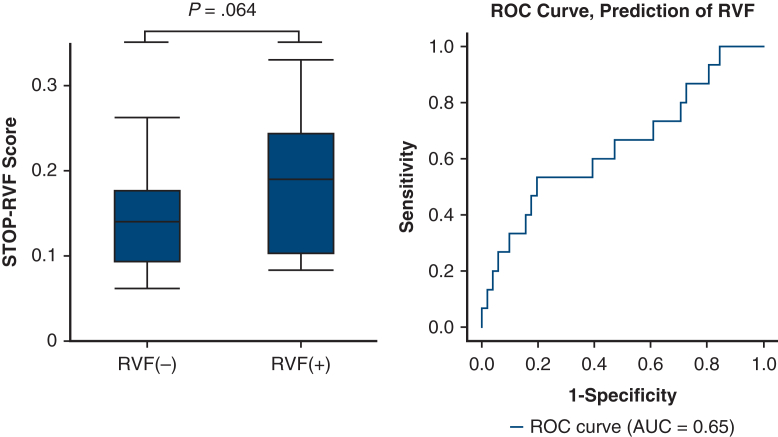
Relationship between STOP-RVF score and right ventricular failure (RVF) in the test data. The STOP-RVF score was applied to the test data patients, but its area under the curve value of 0.65 did not match the performance of the ensemble learning model.

To compare, we calculated risk scores for our test data using the STOP-RVF risk calculator. Patients in the RVF group exhibited a significantly higher tendency for elevated risk scores; however, when applied solely to our test data, the STOP-RVF calculator yielded an AUC of 0.65, which was lower than that achieved by our proposed model (Figure E5).

## Discussion

The highlights of our study can be summarized as follows. Thirteen important medically significant variables suitable for prediction were selected: sex, age, non-ICM, BSA, BUN, AST, LVDd, left ventricular ejection fraction, CO, RVSWI, CVP/PCWP, PAPi, and INTERMACS profile 1 or 2. In predicting the risk of RVF, the AUC, accuracy, and F1 score identified ensemble learning as the most reliable among the 5 ML algorithms. Ensemble learning using 13 variables accurately predicted the risk of RVF, achieving an AUC of 0.888 (95% CI, 0.756-0.975), accuracy of 0.879 (95% CI, 0.788-0.940), F1 score of 0.750 (95% CI, 0.546-0.889), and sensitivity of 0.800 (95% CI, 0.579-1.000). Patients classified into the RVF low-risk group by our algorithm exhibited favorable long-term outcomes, with 0.5-, 1-, 2-, and 3-year survival rates of 93.2%, 93.2%, 90.5%, and 90.5%, respectively.

RVF occurs in 15% to 40% of patients who undergo LVAD implantation and is considered a risk factor for deleterious conditions and poor outcomes.^3^^,^^5^^,^^6^^,^^11^ The complex mechanisms of RVF make it difficult to predict its occurrence after LVAD implantation using traditional linear multivariable analyses.^4^ While traditional regression models are foundational in cardiovascular research, ML was selected because of its demonstrated potential to achieve high predictive performance in complex clinical risk stratification in recent years.^12^^,^^18^ In the field of ML, variable selection is widely performed and can improve model prediction performance while reducing measurement and storage requirements.^19^ The present study selected 13 important variables and performed a risk assessment using ensemble learning, a popular supervised ML algorithm.

Our results are clinically important. Here the selection of important variables showed that a small left ventricular cavity, non-ICM etiology, elevated CVP/PCWP ratio, and decreased PAPi level were significant risk factors for RVF.^4^ These findings suggest that patients who demonstrate concomitant increases in right ventricular and left ventricular stiffness, which elevates CVP/PCWP and decreases PAPi, are at high risk of RVF after LVAD implantation. This result aligns with the right ventricle's function as a low-contractile “volume pump” described by the Frank-Starling mechanism.^20^ Our study also demonstrates that a poor preoperative conditions, such as an INTERMACS profile of 1 or 2, contributes to postoperative RVF, suggesting the importance of preoperative management.

We evaluated our model’s performance by examining the relationship between RVF risk and all-cause mortality in our test data. Our ML model predicted a tendency toward worse long-term outcomes for the high-RVF-risk group, indicating the clinical significance of its predictions. We also computed risk scores for the test data using the STOP-RVF risk calculator. Although the RVF group showed a significant tendency toward higher risk scores, its AUC was 0.65, which is lower than that of our model (Figure E5). There are several reasons for this finding. First, the exclusion of patients requiring preoperative extracorporeal membrane oxygenation potentially limited the representation of the patient population for which the STOP-RVF score was optimized. Second, our model includes such parameters as BSA and LVDd, which represent heart chamber size, enabling us to appropriately reflect the risk in patients with smaller left ventricular size. Furthermore, ML’s proficiency in analyzing interrelated and nonlinear variables facilitated the accurate prediction of RVF risk in individual patients. In this study, RVSWI was not a significant risk factor for RVF; however, it exhibited a bimodal distribution in which both high and low values were associated with increased risk, suggesting that incorporating this factor could improve predictive accuracy.

**Figure E6:**
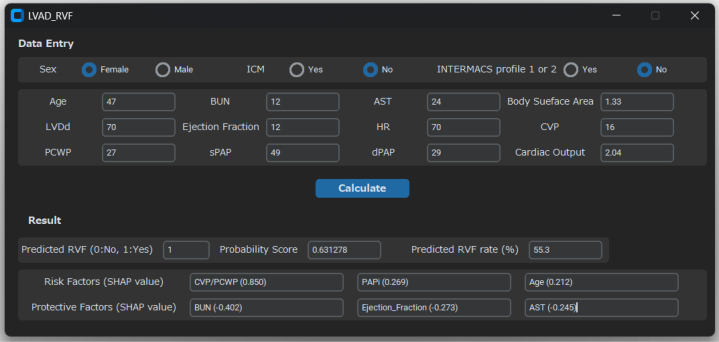
A user-friendly clinical decision support application enabling individualized right ventricular failure (RVF) risk estimation and Shapley additive explanations (SHAP)-based visualization of patient-specific risk factors without requiring programming expertise. *ICM*, ischemic cardiomyopathy; *INTERMACS*, Interagency Registry for Mechanically Assisted Circulatory Support; *BUN*, blood urea nitrogen; *AST*, aspartate aminotransferase; *LVDd*, left ventricular end-diastolic dimension; *HR*, heart rate; *CVP*, central venous pressure; *PCWP*, pulmonary capillary wedge pressure; *PAP*, pulmonary artery pressure.

Medical research using ML is often perceived as a “black box” involving an unclear decision making process. However, in this study, by using SHAP values, we were able to discern the degree to which each factor influenced an individual patient’s outcomes as well as the model overall. This method is highly useful, as it ensures the model’s medical validity. Furthermore, by visualizing the factors that influence individual patient outcomes, the method can help optimize preoperative management and risk assessments. To promote real-world clinical adoption, we also developed a user-friendly application that enables clinicians to use the ML model without any programming knowledge (Figure E6).

## Limitations

Our study has several limitations. First, it was a retrospective study that included a limited number of patients with RVF. Second, 6 different LVAD types were included, given the varying availability of each during the study period, further limiting the number of patients using each device. Third, although no significant difference in RVF incidence was observed between the included patients and excluded patients (22.0% vs 25.0%; *P* = .544), potential selection bias related to the exclusion of patients with missing data cannot be fully excluded, as a detailed comparison of baseline characteristics was limited. Finally, some variables were excluded because of missing data; thus, we performed a 5-fold cross-validation in the model evaluation to minimize bias.

## Conclusion

This study successfully demonstrates that supervised ML can accurately predict RVF and identify its unique risk factors in individual patients following LVAD implantation. By leveraging SHAP values, our predictive model not only offers precise risk assessment but also provides valuable insights into the specific factors influencing each patient's outcome. This interpretability allows for the preoperative identification of LVAD patients at high risk of developing RVF, paving the way for more optimized and personalized clinical management.

## Figures and Tables

**Table E1:** Summary of model performance

**Parameter**	**AUC (95% CI)**	**Accuracy (95% CI)**	**F1 score (95% CI)**	**PPV (95% CI)**	**NPV (95% CI)**	**Sensitivity (95% CI)**	**Specificity (95% CI)**
**Ensemble learning**	0.89 (0.76-0.98)	0.88 (0.79-0.94)	0.75 (0.55-0.89)	0.71 (0.47-0.91)	0.94 (0.87-1.00)	0.80 (0.58-1.00)	0.90 (0.82-0.98)
**Random forest**	0.84 (0.68-0.95)	0.80 (0.70-0.89)	0.67 (0.45-0.81)	0.54 (0.33-0.72)	0.95 (0.88-1.00)	0.87 (0.67-1.00)	0.78 (0.67-0.88)
**XGBoost**	0.88 (0.76-0.97)	0.82 (0.73-0.91)	0.68 (0.48-0.83)	0.57 (0.36-0.76)	0.95 (0.88-1.00)	0.87 (0.67-1.00)	0.80 (0.70-0.90)
**SVM**	0.61 (0.42-0.79)	0.76 (0.65-0.85)	0.47 (0.22-0.67)	0.47 (0.21-0.73)	0.84 (0.74-0.94)	0.47 (0.20-0.73)	0.84 (0.74-0.94)
**Logistic regression**	0.80 (0.65-0.92)	0.77 (0.67-0.88)	0.57 (0.34-0.76)	0.50 (0.28-0.73)	0.89 (0.79-0.98)	0.67 (0.40-0.89)	0.80 (0.69-0.90)

*AUC*, Area under the curve; *CI*, confidence interval; *PPV*, positive predictive value; *NPV*, negative predictive value; *XGBoost*, eXtreme Gradient Boosting; *SVM*, support vector machines.

## References

[1] Kormos R.L., Cowger J., Pagani F.D. (2019). The Society of Thoracic Surgeons Intermacs Database Annual Report: evolving indications, outcomes, and scientific partnerships. J Heart Lung Transplant.

[2] Starling R.C., Naka Y., Boyle A.J. (2011). Results of the post-U.S. Food and Drug Administration-Approval Study with a continuous flow left ventricular assist device as a bridge to heart transplantation: a prospective study using the INTERMACS (Interagency Registry for Mechanically Assisted Circulatory Support). J Am Coll Cardiol.

[3] Dang N.C., Topkara V.K., Mercando M. (2006). Right heart failure after left ventricular assist device implantation in patients with chronic congestive heart failure. J Heart Lung Transpl.

[4] Kiernan M.S., Grandin E.W., Brinkley M. (2017). Early right ventricular assist device use in patients undergoing continuous-flow left ventricular assist device implantation: incidence and risk factors from the interagency registry for mechanically assisted circulatory support. Circ Heart Fail.

[5] Lampert B.C., Teuteberg J.J. (2015). Right ventricular failure after left ventricular assist devices. J Heart Lung Transpl.

[6] Matthews J.C., Koelling T.M., Pagani F.D., Aaronson K.D. (2008). The right ventricular failure risk score a pre-operative tool for assessing the risk of right ventricular failure in left ventricular assist device candidates. J Am Coll Cardiol.

[7] Samura T., Yoshioka D., Asanoi H. (2019). Right atrial pressure waveform predicts right ventricular failure after left ventricular assist device implantation. Ann Thorac Surg.

[8] Fitzpatrick J.R., Frederick J.R., Hiesinger W. (2009). Early planned institution of biventricular mechanical circulatory support results in improved outcomes compared with delayed conversion of a left ventricular assist device to a biventricular assist device. J Thorac Cardiovasc Surg.

[9] Fitzpatrick J.R., Frederick J.R., Hsu V.M. (2008). Risk score derived from pre-operative data analysis predicts the need for biventricular mechanical circulatory support. J Heart Lung Transpl.

[10] Kang G., Ha R., Banerjee D. (2016). Pulmonary artery pulsatility index predicts right ventricular failure after left ventricular assist device implantation. J Heart Lung Transpl.

[11] Kormos R.L., Teuteberg J.J., Pagani F.D. (2010). Right ventricular failure in patients with the HeartMate II continuous-flow left ventricular assist device: incidence, risk factors, and effect on outcomes. J Thorac Cardiovasc Surg.

[12] Motwani M., Dey D., Berman D.S. (2017). Machine learning for prediction of all-cause mortality in patients with suspected coronary artery disease: a 5-year multicentre prospective registry analysis. Eur Heart J.

[13] Samad M.D., Ulloa A., Wehner G.J. (2019). Predicting survival from large echocardiography and electronic health record datasets: optimization with machine learning. JACC Cardiovasc Imaging.

[14] Taleb I., Kyriakopoulos C.P., Fong R. (2024). Machine learning multicenter risk model to predict right ventricular failure after mechanical circulatory support: the STOP-RVF Score. JAMA Cardiol.

[15] Scott M., Lundberg S.-I.L. (2017). Proceedings of the 31st International Conference on Neural Information Processing Systems.

[16] Elreedy D., Atiya A.F. (2019). A Comprehensive Analysis of Synthetic Minority Oversampling Technique (SMOTE) for handling class imbalance. Inform Sci.

[17] García V., Sánchez J.S., Mollineda R.A. (2010). Exploring the performance of resampling strategies for the class imbalance problem. Lect Notes Artif Int.

[18] Harada D., Aasanoi H., Ushijima R. (2018). Impact of right ventricular distensibility on congestive heart failure with preserved left ventricular ejection fraction in the elderly. Heart Vessels.

[19] Samad M.D., Wehner G.J., Arbabshirani M.R. (2018). Predicting deterioration of ventricular function in patients with repaired tetralogy of Fallot using machine learning. Eur Heart J Cardiovasc Imaging.

[20] Karunanithi M.K., Michniewicz J., Copeland S.E., Feneley M.P. (1992). Right ventricular preload recruitable stroke work, end-systolic pressure-volume, and dP/dtmax-end-diastolic volume relations compared as indexes of right ventricular contractile performance in conscious dogs. Circ Res.

